# Clinical and CT Imaging Features of Chronic Pancreatitis: A Cross‐Sectional Study From Vietnam

**DOI:** 10.1002/jgh3.70430

**Published:** 2026-06-07

**Authors:** Tran Thi Luong Vo, Nguyen Phuoc Ma, Dung Dang Quy Ho, Hoang Huu Bui

**Affiliations:** ^1^ Department of Internal Medicine and Gastro‐Hepato Integrated Research Team (GHIRT‐002.TCM2025), School of Medicine University of Medicine and Pharmacy Ho Chi Minh City Vietnam; ^2^ Department of Gastroenterology Cho Ray Hospital Ho Chi Minh City Vietnam; ^3^ Department of Endoscopy Cho Ray Hospital Ho Chi Minh City Vietnam; ^4^ Department of Gastroenterology University Medical Center Ho Chi Minh City Vietnam

**Keywords:** chronic pancreatitis, clinical characteristics, CT imaging

## Abstract

**Aims:**

Chronic pancreatitis (CP) is a progressive inflammatory condition with insidious and nonspecific symptoms; however, data on its clinical and computed tomography (CT) based characteristics in Vietnam remain limited. This study aimed to characterize the clinical features and contrast‐enhanced CT findings of Vietnamese patients with CP and to assess the association between clinical manifestations and morphological severity using the Cambridge classification.

**Methods and Results:**

We conducted a cross‐sectional study of patients diagnosed with CP at a tertiary hospital in Ho Chi Minh City, Vietnam. Demographics, risk factors, clinical symptoms, and CT imaging characteristics were recorded. Morphologic severity was graded using the Cambridge system. A total of 160 patients were included; 85.6% were male, with a median age of 50 years. Alcohol‐related disease was the predominant etiology (62.5%). Notably, 24.4% had disease onset before 35 years of age, and 33.1% had no prior history of acute pancreatitis. Abdominal pain was the most common symptom (87.5%), followed by weight loss and diabetes mellitus. CT imaging demonstrated advanced structural abnormalities, with pancreatic calcifications in 85.0% of patients, ductal dilatation in 73.1%, and 92.5% classified as Cambridge grade 4. Within this predominantly advanced‐stage cohort, no statistically significant association was detected between clinical manifestations and CT‐based morphological severity.

**Conclusion:**

In this tertiary‐center CT‐based cohort from Vietnam, CP was characterized by heterogeneous clinical manifestations and predominantly advanced CT abnormalities. The absence of a detectable symptom–severity association in this advanced‐stage cohort supports the need for more comprehensive diagnostic approaches to enable earlier recognition of CP.

**Abbreviations**
ACP: alcohol‐related chronic pancreatitis
AP: acute pancreatitis
BMI: body mass index
CP: chronic pancreatitis
CT: computed tomography
DM: diabetes mellitus
ICP: idiopathic chronic pancreatitis
IQR: interquartile range
MPD: main pancreatic duct
SCP: smoking‐associated chronic pancreatitis

## 1 Introduction

Chronic pancreatitis (CP) is a progressive fibro‐inflammatory disease of the pancreas characterized by irreversible structural remodeling and gradual loss of exocrine and endocrine function. Its chronic symptoms and long‐term complications markedly impair quality of life and are associated with poor overall prognosis [1, 2]. Worldwide, the incidence and prevalence of CP continue to rise, reflecting an increasing healthcare burden and the pressing need for earlier diagnosis and more effective management [3].

As a chronic condition that evolves gradually and often presents with subtle or nonspecific symptoms, CP is frequently recognized only after substantial and irreversible pancreatic injury has occurred [3]. Its clinical manifestations commonly overlap with those of other gastrointestinal disorders, creating diagnostic uncertainty and limiting the reliability of clinical evaluation alone. A detailed understanding of the clinical characteristics of CP is therefore essential to support diagnosis, monitor disease progression, and guide risk stratification. However, systematic data describing the clinical profile of CP in Vietnam remain scarce.

Diagnostic evaluation is further challenged by the limited practicality and sensitivity of histologic assessment and pancreatic function testing, particularly in early disease [4]. As a result, cross‐sectional imaging has become an indispensable component of the diagnostic pathway for CP. In Vietnam, this reliance on imaging is amplified by limited access to advanced modalities such as endoscopic ultrasound (EUS) and magnetic resonance cholangiopancreatography (MRCP) because of their high cost and restricted availability, as well as the invasive nature of EUS. Consequently, contrast‐enhanced computed tomography (CT) serves as the principal modality for confirming the diagnosis, assessing structural severity, detecting complications, and guiding management. Despite its central role, comprehensive data on CT imaging features and their relationship to clinical presentation in Vietnamese patients with CP remain limited.

Accordingly, this study was designed to comprehensively characterize the clinical manifestations and CT‐based structural features of CP in Vietnamese patients, providing essential baseline evidence to improve diagnostic accuracy and inform earlier detection and management strategies.

## 2 Methods

### 2.1 Study Design and Data Source

The present study was a cross‐sectional analysis of prospectively collected data from the same patient cohort used in our previous publication, “*PRSS1*, *SPINK1* Mutations and Associated Factors in Vietnamese Patients with Chronic Pancreatitis” (JGH Open 2025;9(9):e70275. doi:10.1002/jgh3.70275). The previous study focused on *PRSS1* and *SPINK1* mutations, whereas the current analysis examines clinical and CT imaging characteristics. Data were collected prospectively at Cho Ray Hospital, a tertiary referral center in Ho Chi Minh City, Vietnam, between October 2022 and December 2024, under approval from the same institutional ethics committee (ID: 756/HDDD‐DHYD, October 2022), with written informed consent obtained from all participants. Because this was a secondary cross‐sectional analysis of an existing prospectively collected cohort, no additional patient contact was required.

### 2.2 Study Population

Eligible participants were adults (≥ 18 years) diagnosed with CP according to the 2020 American College of Gastroenterology (ACG) Clinical Guidelines [5]. In accordance with these recommendations, patients with recognized risk factors, such as chronic alcohol use or smoking, and/or characteristic clinical manifestations, including recurrent or chronic abdominal pain, steatorrhea, symptoms of pancreatic insufficiency, or a history of recurrent acute pancreatitis (RAP), underwent contrast‐enhanced abdominal computed tomography (CT) as the first‐line imaging modality. Patients were eligible for inclusion if CT demonstrated morphologic abnormalities consistent with CP, defined as Cambridge grade ≥ 2 [6]. In this study, the use of CT reflected its routine availability and standardized application in clinical practice at our tertiary referral hospital. Nevertheless, reliance on CT and the Cambridge classification may have reduced sensitivity for detecting early‐stage CP and subtle morphological changes.

Patients were excluded if they met any of the following conditions: (1) pregnancy at the time of enrollment; (2) advanced systemic comorbidities that could interfere with clinical assessment or confound CT interpretation (such as decompensated cirrhosis, end‐stage renal disease, or severe cardiac disease); (3) known active malignancy before enrollment, except for pancreatic cancer newly identified during the same hospitalization for CP evaluation; or (4) known contraindications to iodinated contrast agents.

### 2.3 Data Collection

Clinical data were obtained through structured patient interviews and review of medical records. Collected variables included demographic characteristics, admission presentation, history of AP with the number of prior episodes, comorbid conditions, and exposure to recognized risk factors such as alcohol and tobacco use. Symptom onset and principal clinical features of CP, including abdominal pain, steatorrhea, weight loss, and diabetes mellitus (DM), as well as other associated symptoms and complications, were documented. Pancreatic cancer was recorded when documented during the same hospitalization in which patients were evaluated for CP, based on medical record review, clinical assessment, CT findings, and histopathological confirmation where available. It was not considered an incident outcome detected during subsequent follow‐up. Details of prior endoscopic and surgical interventions were obtained from medical records. Endoscopic interventions included biliary and/or pancreatic endotherapy (e.g., ERCP‐based sphincterotomy, stenting, or stone extraction). Surgical interventions referred to operations for CP‐related symptoms or complications, such as ductal obstruction, pseudocysts, or persistent pain. All treatment decisions were made by the treating clinical team as part of routine care and were not determined by the study protocol.

Alcohol use was defined as lifetime consumption of ≥ 20 standard drinks (≥ 280 g ethanol), with weekly intake classified according to NAPS2 as moderate (4–7 drinks/week for women; 4–14 drinks/week for men), heavy (8–34 drinks/week for women; 15–34 drinks/week for men), or very heavy (≥ 35 drinks/week) [7].

Smoking status was classified as never smokers (< 100 cigarettes in a lifetime), former smokers, or current smokers. Cumulative exposure was quantified in pack‐years when available [8]. Age at onset was defined as the age at the earliest clinical manifestation, including the first presentation of abdominal pain, the first episode of AP, or the first manifestation of exocrine or endocrine insufficiency.

RAP was defined as two or more documented episodes of AP meeting the Revised Atlanta criteria. Acute exacerbation was defined as an episode of AP occurring in a patient with established CP.

Etiologic classification of CP was based on the presumed primary clinical etiology. Alcohol‐related CP (ACP) was defined as sustained alcohol consumption exceeding 80 g/day for men or 60 g/day for women for at least 2 years. When alcohol and smoking exposures coexisted, patients meeting criteria for ACP were classified as ACP, while smoking was recorded as a coexisting risk factor. Smoking‐associated CP (SCP) was assigned only to patients with tobacco exposure ≥ 30 pack‐years in the absence of alcohol‐related or other identifiable etiologies. Idiopathic CP (ICP) was defined as the absence of an identifiable primary clinical etiology despite appropriate clinical evaluation. Genetic variants identified in our previous study were considered coexisting susceptibility factors and were not used as standalone criteria for assigning hereditary CP [9, 10].

### 2.4 Imaging Assessment

All participants underwent contrast‐enhanced abdominal CT at the time of diagnosis using a standardized multiphasic pancreatic protocol. Two experienced abdominal radiologists, blinded to all clinical information, independently reviewed each CT scan, with discrepancies resolved by consensus. Morphologic evaluation followed a CT‐adapted Cambridge classification, grading pancreatic abnormalities from 0 to 4. The imaging features systematically analyzed and reported in this study included main pancreatic duct (MPD) dilatation or irregularity, parenchymal or ductal calcifications, pseudocysts, and intraductal stones. Anteroposterior diameters of the pancreatic head, body, and tail were measured to describe pancreatic morphology; however, pancreatic atrophy was not used to assign Cambridge grade. The MPD diameter was measured on portal venous phase images at the pancreatic head, body, and tail using the maximal inner‐to‐inner luminal distance, and the largest value obtained was recorded for analysis. Pancreatic cyst size was assessed on the axial slice demonstrating the maximal cyst dimension.

### 2.5 Statistical Analysis

Descriptive statistics were used to summarize all clinical and imaging variables. Continuous variables were summarized as mean ± SD or median (IQR) and compared using the *T*‐test, Mann–Whitney U test, ANOVA, or Kruskal–Wallis test, as appropriate. Categorical variables were compared using the *χ* ^2^ or Fisher's exact test. Statistical significance was set at *p* < 0.05.

## 3 Results

### 3.1 Baseline Characteristics

Among the 160 included patients, males accounted for 85.6%, with a median age of 50 years (IQR 38–60). The median BMI was 19.3 kg/m^2^ (IQR 16.9–21.4). Comorbidities were present in some patients, with hypertension being the most common (23.7%). Alcohol consumption was documented in 65.6% of patients, 57.1% of whom were active drinkers. Among ever drinkers, very heavy intake was the most common pattern (69.5%), and 40.0% had consumed alcohol for 20–30 years. Smoking exposure was similarly prevalent, with 66.9% classified as ever smokers; among them, 72.0% were current smokers (Table 1). Regarding etiology, ACP accounted for 62.5% of cases, while ICP represented 31.9% and SCP 5.6%. A history of AP was present in 66.9% of patients (median 1 episode; IQR 0–4). Notably, one‐third (33.1%) had no prior acute episode, indicating that CP may develop in the absence of preceding AP (Table 2).

**TABLE 1:** Alcohol consumption and smoking status among patients with chronic pancreatitis.

Characteristics	Number of patients (*n*)	Percentage (%)
Alcohol consumption (*n* = 160)		
Ever drinkers	105	65.6
Abstainer	55	34.4
Among ever drinkers (*n* = 105)		
Current drinkers	60	57.1
Former drinkers	45	42.9
Drinking patterns		
Moderate	8	7.7
Heavy	24	22.8
Very heavy	73	69.5
Duration of alcohol consumption (years)		
< 20	44	41.9
20–30	42	40.0
> 30	19	18.1
Tobacco smoking (*n* = 160)		
Non‐smokers	53	33.1
Ever smokers	107	66.9
Current smokers	77	72.0
Former smokers	30	28.0
Cumulative amount of smoking (pack‐years)		
< 20	51	47.6
20–30	31	29.0
> 30	25	23.4

**TABLE 2:** Medical history, age at disease onset, and initial presenting symptoms of chronic pancreatitis.

Characteristics	Total (*n* = 160)	ACP (*n* = 100)	SCP (*n* = 9)	ICP (*n* = 51)	*p*
History of acute pancreatitis					
Prior acute pancreatitis, *n* (%)	107 (66.9)	70 (70.0)	4 (44.4)	33 (64.7)	0.3
Number of previous acute pancreatitis episodes	1.0 (0–4.0)	1.0 (0–4.0)	0.0 (0.0–3.0)	1.0 (0–4.0)	0.6
Age at onset (years)					
Mean age of onset (years)	44 ± 15	46 ± 12	62 ± 8	39 ± 18	< 0.001
Early‐onset (before 35 years‐old), *n* (%)	39 (24.4)	16 (16.0)	0 (0)	23 (45.1)	< 0.001
Initial presenting symptoms					
Abdominal pain, *n* (%)	119 (74.4)	75 (75)	5 (55.6)	39 (76.5)	0.4
Steatorrhea *n* (%)	0	0	0	0	—
Diabetes mellitus, *n* (%)	38 (23.8)	24 (24.0)	3 (33.3)	10 (19.6)	0.6
First episode of acute pancreatitis, *n* (%)	13 (8.1)	9 (9.0)	2 (22.2)	2 (3.9)	0.14

*Note:* Data are presented as the median (25%–75%); *n* (%).

Abbreviations: ACP: alcohol‐related chronic pancreatitis; ICP: idiopathic chronic pancreatitis; SCP: smoking‐associated chronic pancreatitis.

### 3.2 Clinical Features

The mean age at onset was 44 ± 15 years, with early‐onset disease (< 35 years) in 24.4% and significantly more frequent among idiopathic cases (*p* < 0.001). The most common initial symptom was abdominal pain (74.4%), followed by new‐onset DM (23.8%). Additionally, 8.1% were first diagnosed with CP during an episode of AP.

Abdominal pain was also the predominant symptom during the disease course (87.5%). Weight loss was the next most common symptom (61.8%), with a median 6 kg reduction over the preceding 6 months (IQR 4–10), followed by DM (46.9%) and bloating (40.6%). Steatorrhea (8.1%) and jaundice (6.3%) were uncommon, although jaundice occurred significantly more often in ACP (Table 3).

**TABLE 3:** Clinical manifestations, complications, and interventions in patients with chronic pancreatitis.

Characteristics	Total (*n* = 160)	ACP (*n* = 100)	SCP (*n* = 9)	ICP (*n* = 51)	*p*
Clinical features					
Abdominal pain, *n* (%)	140 (87.5)	86 (86.0)	7 (78.0)	47 (92.1)	0.3
Weight loss, *n* (%)	99 (61.8)	67 (67.0)	6 (66.6)	26 (50.9)	0.2
Diabetes mellitus, *n* (%)	75 (46.9)	48 (48)	4 (44.4)	23 (45.1)	0.9
Acute exacerbation, *n* (%)	66 (41.2)	44 (44.0)	5 (55.5)	17 (33.3)	0.3
Abdominal bloating, *n* (%)	65 (40.6)	38 (38.0)	4 (44.4)	23 (45.1)	0.7
Steatorrhea, *n* (%)	13 (8.1)	8 (8.0)	1 (11.1)	4 (7.8)	0.8
Jaundice, *n* (%)	10 (6.3)	10 (10.0)	0 (0.0)	0 (0.0)	0.042
Complications					
Hemorrhage, *n* (%)	2 (1.3)	2 (2.0)	0 (0.0)	0 (0.0)	0.6
Pseudoaneurysm, *n* (%)	5 (3.1)	5 (5.0)	0 (0.0)	0 (0.0)	0.4
Biliary obstruction, *n* (%)	29 (18.1)	23 (23.0)	2 (22.2)	4 (7.8)	0.047
Fistula, *n* (%)	6 (3.8)	5 (5.0)	0 (0.0)	1 (2.0)	0.4
Splenic vein thrombosis, *n* (%)	18 (11.2)	13 (13.0)	0 (0.0)	5 (9.8)	0.7
Pancreatic cancer^a^, *n* (%)	6 (3.8)	5 (5.0)	0 (0.0)	1 (2.0)	0.8
Previous interventions					
Endoscopic intervention, *n* (%)	26 (16.3)	14 (14.0)	0 (0.0)	12 (23.5)	0.2
Surgical intervention, *n* (%)	29 (18.1)	14 (14.0)	1 (11.1)	14 (27.5)	0.11

*Note:* The data are presented as *n* (%).

Abbreviations: ACP: alcohol‐related chronic pancreatitis; ICP: idiopathic chronic pancreatitis; SCP: smoking‐associated chronic pancreatitis.

^a^ Pancreatic cancer refers to cases diagnosed during the same hospitalization in which patients were evaluated for CP, not cancers detected during subsequent follow‐up.

### 3.3 Complications and Treatment

Complications were documented in some patients. Biliary obstruction was the most frequent complication (18.1%) and occurred significantly more often in ACP. Vascular complications were identified in 11.2% of patients, including splenic, portal, and other splanchnic vein thromboses. Pancreatic fistulas and pseudoaneurysms were observed in 3.8% and 3.1% of patients, respectively, whereas hemorrhagic events were rare (1.3%). Pancreatic cancer was diagnosed during the same hospitalization for CP evaluation in 6 patients (3.8%) and was therefore recorded as a concomitant diagnosis rather than an incident follow‐up outcome. Apart from biliary obstruction, the distribution of these findings did not differ significantly across etiologic groups (Table 3).

A subset of patients had a history of interventional treatment: 26 (16.3%) had undergone endoscopic procedures, and 29 (18.1%) had undergone surgery for CP‐related symptoms or complications. No statistically significant differences in intervention rates were detected across etiologic groups (Table 3).

### 3.4 Imaging Features

The median MPD diameter was 6.0 mm (IQR 4.0–9.0); MPD dilatation > 4 mm was present in 73.1% of patients, borderline duct caliber of 2–4 mm in 15.6%, and normal duct caliber < 2 mm in 11.2%, with no significant differences across etiologic groups. Pancreatic pseudocysts were detected in 22.5% of patients (median size 23.0 mm), and 91.6% measured ≥ 10 mm; prevalence and size were similar among etiologies. Pancreatic duct stones were present in 62.5% of patients, and parenchymal calcifications in 85.0%, with no statistically significant variation by etiology. According to the Cambridge classification, most patients (92.5%) were categorized as grade 4, and distribution did not differ between etiologic groups (Table 4).

**TABLE 4:** Abdominal computed tomography characteristics of chronic pancreatitis.

CT characteristics	Total (*n* = 160)	ACP (*n* = 100)	SCP (*n* = 9)	ICP (*n* = 51)	*p*
Main pancreatic duct diameter (mm)	6.0 (4.0–9.0)	7.0 (4.0–9.0)	7.0 (5.0–8.0)	6.0 (3.9–8.0)	0.3
Categorization of main pancreatic duct diameter					0.3
< 2 mm, *n* (%)	18 (11.2)	8 (8.0)	2 (22.2)	8 (15.6)	
2–4 mm, *n* (%)	25 (15.6)	18 (18.0)	0 (0.0)	7 (13.7)	
> 4 mm, *n* (%)	117 (73.1)	74 (74.0)	7 (77.7)	36 (70.5)	
Pancreatic cysts					
Presence of cysts, *n* (%)	36 (22.5)	28 (28.0)	2 (22.2)	6 (11.7)	0.065
Cyst diameter (mm)	23 (17–36)	23 (17–33)	44 (31–56)	30 (16–38)	0.8
Categorization of pancreatic cyst diameter					
< 10 mm, *n* (%)	3 (8.3)	2 (7.1)	0 (0.0)	1 (16.6)	0.5
≥ 10 mm, *n* (%)	33 (91.6)	26 (92.8)	2 (100)	5 (83.3)	
Pancreatic ductal stones and calcifications					
Duct stones, *n* (%)	100 (62.5)	66 (66.0)	5 (55.5)	29 (56.8)	0.5
Calcification, *n* (%)	136 (85.0)	88 (88.0)	9 (100)	39 (76.4)	0.1
CT features according to the Cambridge classification					
Cambridge 2, *n* (%)	6 (3.8)	4 (4.0)	0 (0.0)	2 (3.9)	0.9
Cambridge 3, *n* (%)	6 (3.8)	4 (4.0)	0 (0.0)	2 (3.9)	
Cambridge 4, *n* (%)	148 (92.5)	92 (92.0)	9 (100)	47 (92.1)	

*Note:* Data are presented as the median (25%–75%); *n* (%).

Abbreviations: ACP: alcohol‐related chronic pancreatitis; ICP: idiopathic chronic pancreatitis; SCP: smoking‐associated chronic pancreatitis.

### 3.5 Association Between Clinical Features and Imaging Characteristics in Patients With Chronic Pancreatitis

Our study showed that in this predominantly advanced‐stage CT cohort, the frequencies of clinical manifestations did not differ significantly across Cambridge categories (Table 5). However, this finding should be interpreted cautiously given the highly imbalanced distribution of Cambridge grades.

**TABLE 5:** Association between clinical characteristics and abdominal computed tomography characteristics.

Characteristics	CT findings according to the Cambridge classification				*p*
	Total *n* (%) (*n* = 160)	Cambridge 2 *n* (%) (*n* = 6)	Cambridge 3 *n* (%) (*n* = 6)	Cambridge 4 *n* (%) (*n* = 148)	
Abdominal pain	140 (87.5)	6 (100.0)	6 (100.0)	128 (86.5)	0.9
Steatorrhea	13 (8.1)	0 (0.0)	1 (16.7)	12 (8.1)	0.7
Jaundice	10 (6.3)	0 (0.0)	1 (16.7)	9 (6.1)	0.6
Weight loss	99 (61.8)	4 (66.7)	2 (33.3)	93 (62.8)	0.4
Bloating	65 (40.6)	3 (50.0)	2 (33.3)	60 (40.5)	0.9
Diabetes mellitus	75 (46.9)	2 (33.3)	1 (16.7)	72 (48.6)	0.3

*Note:* The data are presented as *n* (%).

## 4 Discussion

This study provides a detailed description of clinical manifestations and CT‐based structural abnormalities in a tertiary‐center cohort of Vietnamese patients with CP. Within this predominantly advanced‐stage CT cohort, we observed no statistically significant associations between clinical features and Cambridge imaging stages.

Heavy and long‐term alcohol consumption, tobacco use, and genetic susceptibility are well‐established contributors to CP, although their relative importance varies across populations. In our cohort, ACP predominated, SCP was less frequent, and nearly one‐third of cases were classified as ICP. Similar patterns have been reported in South Korea and several Western populations, whereas studies from China, Japan, and India have described lower proportions of ACP and higher rates of ICP [11]. These regional differences may reflect variations in alcohol and tobacco exposure, genetic susceptibility, environmental factors, and access to advanced diagnostic modalities that facilitate identification of hereditary, obstructive, or structural causes.

The relatively low proportion of SCP compared with the high prevalence of smoking exposure reflects our presumed primary clinical‐etiology approach. Therefore, the reported frequency of SCP represents cases in which smoking was assigned as the presumed primary clinical etiology and should not be interpreted as the overall prevalence of tobacco exposure in the cohort. Similarly, although pathogenic mutations were identified in 40% of patients in our previous genetic analysis of the same cohort, these findings were considered susceptibility factors or disease modifiers rather than definitive evidence of hereditary CP. Therefore, the reported 30% frequency of ICP reflects cases without an identifiable primary clinical etiology and should be interpreted in the context of CP as a multifactorial disease involving overlapping toxic‐metabolic, genetic, environmental, and other contributing factors.

The male predominance in our cohort aligns with global patterns, reflecting higher alcohol and tobacco exposure among men [12, 13, 14, 15]. The median age of 50 years is comparable to Western studies, where CP is typically diagnosed in mid‐adulthood after long‐standing subclinical progression [13, 15, 16]. In contrast, younger onset reported in China and India likely reflects a greater contribution of idiopathic or hereditary forms [12, 14].

Recurrent AP is well recognized as part of the continuum leading to CP. Consistent with reports from Hungary and the United States, over half of our patients had experienced at least one prior acute episode, whereas markedly lower rates reported in Japan likely reflect differences in population characteristics and diagnostic practices [13, 17, 18]. Importantly, one‐third of our cohort had no history of AP, indicating that the absence of prior acute events does not preclude CP. A small subset was diagnosed with CP during a first episode of AP, suggesting that subclinical CP may remain undetected until unmasked by an acute insult.

Abdominal pain was the most common symptom in our cohort, present in nearly 88% of patients, consistent with reports from Asian and Western populations [13, 19, 20, 21]. Pain typically served as the initial presentation and the main reason for hospitalization, although its patterns varied from episodic attacks related to recurrent AP to persistent or intermittent discomfort. Notably, 12% of patients were asymptomatic, in line with international data, emphasizing that the absence of pain does not exclude CP and reinforcing the need for combined clinical, imaging, and biochemical evaluation.

In our cohort, both endocrine and exocrine insufficiency appeared to be clinically underrecognized: nearly half of patients had DM, yet it was not a prominent presenting feature, and steatorrhea—an indicator of exocrine dysfunction—was reported in only 8.1% of cases. These findings, consistent with wide variability across international studies, highlight that metabolic and digestive symptoms are often not dominant manifestations of CP and may be underestimated without targeted assessment [13, 18, 19, 21].

CP in our cohort was notable for vascular complications. Splanchnic venous thrombosis was observed in 11.2% of patients, consistent with recent international reports reflecting improved imaging‐based detection [21]. Pseudoaneurysms, although less common (3.1%), remain clinically significant given their potential for life‐threatening hemorrhage. Biliary obstruction (18.1%) and pancreatic cancer documented during CP evaluation (3.8%) were broadly comparable to international reports, whereas vascular events were clinically notable because of their severity and diagnostic challenges.

Using the Cambridge classification, most patients in our cohort were classified as grade 4, indicating an advanced morphological stage at diagnosis. This pattern contrasts with US data from Sandhu et al., in which earlier‐stage disease was more frequently represented. Consistent with this advanced CT phenotype, pancreatic calcifications were highly prevalent (85%), and ductal dilatation (73%), intraductal stones (63%), and pancreatic cysts (23%) were also common, with rates higher than those reported in several international cohorts [22]. These findings likely reflect both the tertiary referral setting and the CT‐based inclusion criteria, which preferentially captured patients with established structural abnormalities. Future studies should consider adopting more comprehensive frameworks, such as the modified CT‐based scoring system proposed by the NAPS2 cohort [23], to better characterize the full spectrum of CP severity, including early‐stage disease.

The relationship between clinical manifestations and imaging findings in CP remains incompletely defined. Previous studies from Norway have reported associations between structural abnormalities—such as ductal obstruction, pancreatic atrophy, and calcifications—and clinical outcomes, including exocrine insufficiency and weight loss [24, 25]. In our study, no statistically significant association was detected between clinical manifestations and Cambridge grades. This finding should be interpreted cautiously because imaging severity was highly skewed toward Cambridge grade 4, likely reflecting the tertiary referral setting and CT‐based inclusion criteria. The small number of patients with grades 2–3 limited statistical power for comparisons across severity categories. Therefore, the absence of a detectable clinicoradiological association in this advanced‐stage cohort does not exclude such a relationship across the broader CP spectrum, particularly in earlier‐stage disease.

Our study has several limitations. First, it was conducted at a single tertiary center, used a cross‐sectional design, and included a relatively small sample, which may limit the generalizability of the findings and preclude evaluation of disease progression over time. Second, the use of contrast‐enhanced CT as the only imaging modality may have reduced sensitivity for early‐stage CP and preferentially captured patients with advanced structural disease, thereby limiting comparisons across severity categories and requiring cautious interpretation of clinicoradiological associations.

## 5 Conclusion

In this tertiary‐center CT‐based cohort, CP was characterized by male predominance, alcohol‐related etiology, and abdominal pain as the most common presenting symptom. CP was also observed in patients without a documented history of AP, suggesting that the absence of prior acute episodes should not exclude the diagnosis. Contrast‐enhanced CT frequently revealed advanced morphological abnormalities, including pancreatic calcifications, ductal dilatation, and intraductal stones. No statistically significant association between clinical manifestations and CT‐based morphological severity was detected within this predominantly advanced‐stage cohort. These results support an integrated diagnostic approach combining clinical assessment, biochemical evaluation, and appropriate imaging to improve recognition of CP across a broader disease spectrum.

## Funding

The authors have nothing to report.

## Ethics Statement

Ethical approval for this study was obtained from the Board of Ethics in Biomedical Research of the University of Medicine and Pharmacy at Ho Chi Minh City (ID number: 756/HDDD‐DHYD, signed on 20/10/2022).

## Consent

All eligible patients signed a written informed consent form prior to study inclusion.

## Conflicts of Interest

The authors declare no conflicts of interest.

## Data Availability

The data that support the findings of this study are available on request from the corresponding author. The data are not publicly available due to privacy or ethical restrictions.
